# Renin–angiotensin system inhibitor use and survival outcomes in lung cancer: a systematic review and meta-analysis

**DOI:** 10.3389/fphar.2026.1953204

**Published:** 2026-09-21

**Authors:** Yongqian Liao, Jinhai Shen, Yonghe Liao, Yuxuan Lin, Nengyong Zeng

**Affiliations:** 1 Department of Respiratory and Critical Care Medicine, The Second People’s Hospital of Qinzhou, Qinzhou, Guangxi, China; 2 State Key Laboratory of Natural Medicines, China Pharmaceutical University, Nanjing, Jiangsu, China; 3 Center for New Drug Safety Evaluation and Research, China Pharmaceutical University, Nanjing, Jiangsu, China; 4 School of Basic Medicine and Clinical Pharmacy, China Pharmaceutical University, Nanjing, Jiangsu, China; 5 Department of Pharmacy, The Second Affiliated Hospital of Guangxi Medical University, Nanning, Guangxi, China; 6 Department of Pharmacy, Guangxi Hospital Division of the First Affiliated Hospital, Sun Yat-sen University, Nanning, Guangxi, China

**Keywords:** lung cancer, meta-analysis, overall survival, progression-free survival, renin-angiotensin system inhibitors

## Abstract

**Background:**

Renin-angiotensin system inhibitors (RASi) have been associated with favorable survival outcomes across various cancers, but their association with survival in lung cancer remains uncertain. We conducted a systematic review and meta-analysis to evaluate the association between RASi use and survival outcomes in patients with lung cancer.

**Methods:**

PubMed and Embase were searched from inception to 6 July 2026. Pooled hazard ratios (HRs) with 95% confidence intervals (CIs) were calculated using random-effects models. Subgroup and sensitivity analyses, including large-study restricted analysis and precision-effect test and precision-effect estimate with standard error (PET-PEESE) regression, were performed to explore heterogeneity and assess the potential influence of small-study effects.

**Results:**

Twenty-six studies involving 403,423 patients were included. RASi use was associated with longer overall survival (OS; HR = 0.76, 95% CI: 0.70–0.83; *I* ^*2*^ = 82.9%) and progression-free survival (PFS; HR = 0.79, 95% CI: 0.70–0.89; *I* ^*2*^ = 40.9%) in conventional random-effects meta-analyses. Subgroup analyses generally showed consistent associations across treatment modalities and geographic regions, although substantial heterogeneity remained in several subgroups. Egger’s test indicated small-study effects for OS. Although the large-study restricted analysis remained significant (HR = 0.82, 95% CI: 0.74–0.90), PET-PEESE adjustment attenuated the estimate to the null (HR = 1.00, 95% CI: 0.95–1.05). No significant evidence of small-study effects was observed for PFS.

**Conclusion:**

Conventional meta-analysis showed an association between RASi use and longer OS and PFS. However, the OS association was attenuated to the null after adjustment for small-study effects, indicating that the magnitude and robustness of the observed OS association remain uncertain. These findings should therefore be interpreted cautiously and require confirmation in well-designed prospective studies.

**Systematic Review Registration:**

https://www.crd.york.ac.uk/prospero/display_record.php?ID=CRD420251053671, identifier: CRD420251053671.

## Introduction

Lung cancer remains the leading cause of cancer-related mortality worldwide (Leiter et al., 2023). According to GLOBOCAN 2024 estimates, it accounts for approximately 2.64 million new cases and 1.86 million deaths annually, posing a substantial burden on global public health systems (Sung et al., 2026).

Hypertension is a common comorbidity among lung cancer patients, with reported prevalence influenced by heterogeneity in study populations, diagnostic criteria, and treatment contexts (Almatrafi et al., 2023; Cohen et al., 2023; Kidoguchi et al., 2021; Sahni, 2023). This increased prevalence is likely attributable to shared risk factors—including smoking, obesity, and physical inactivity—as well as treatment-related toxicities (Cohen et al., 2023; Kidoguchi et al., 2021; Sahni, 2023; Koene et al., 2016). For example, anti-angiogenic agents (e.g., tyrosine kinase inhibitors) and platinum-based chemotherapy, both widely used in advanced lung cancer, are well-recognized causes of treatment-induced hypertension (Kidoguchi et al., 2021; Sahni, 2023; van Dorst et al., 2021). Consequently, renin–angiotensin system inhibitors (RASi), one of the most frequently prescribed antihypertensive drug classes, are commonly administered as concomitant medications in this patient population.

RASi, comprising angiotensin-converting enzyme inhibitors (ACEi) and angiotensin II (Ang II) receptor blockers (ARB), are established therapies for hypertension and cardiovascular diseases (Alcocer et al., 2023). Beyond their cardiovascular benefits, accumulating evidence suggests that RASi may also influence tumor biology and the tumor microenvironment (George et al., 2010). The renin–angiotensin system (RAS), primarily through Ang II signaling *via* the Ang II type 1 receptor (AT_1_R), has been implicated in multiple hallmarks of cancer, including cell proliferation, angiogenesis, inflammation, extracellular matrix remodeling, and fibrosis (George et al., 2010; Perini et al., 2020). RAS inhibition may modulate these processes by attenuating Ang II-mediated signaling, VEGF-driven angiogenesis, and tumor-associated inflammation and fibrosis (Perini et al., 2020).

While some observational and genetic studies have raised concerns regarding a potential increased risk of lung cancer associated with ACEi exposure, these findings primarily address cancer development rather than prognosis after diagnosis. A recent meta-analysis reported an increased incidence of lung cancer among ACEi users (OR = 1.19, 95% CI: 1.05–1.36) (Wu et al., 2023), and Mendelian randomization evidence has similarly suggested an association between genetically proxied ACE inhibition and lung cancer risk (Yao et al., 2023). The apparent discrepancy between these findings and the favorable survival associations reported in some studies of patients with established lung cancer may reflect differences in the clinical context of exposure, duration of treatment, patient characteristics, and underlying biological mechanisms. Potential explanations include confounding by indication and differences in smoking exposure and other baseline risk factors, as well as the possibility that ACEi-related bradykinin/substance P accumulation may influence processes relevant to carcinogenesis, whereas inhibition of Ang II–AT_1_R signaling may influence tumor progression, angiogenesis, stromal remodeling, and the tumor microenvironment.

The association between RASi use and survival outcomes in patients with lung cancer remains controversial. While several observational studies have reported improved overall survival (OS) and progression-free survival (PFS) among RASi users receiving chemotherapy, targeted therapy, or immunotherapy (Wilop et al., 2009; Hsieh et al., 2025; Shen et al., 2023), others have reported no significant survival benefit (Kichenadasse et al., 2021). These inconsistent findings may reflect substantial heterogeneity in study design, patient characteristics, tumor stage, treatment modality, and definitions of RASi exposure. Furthermore, whether ACEi and ARB are associated with comparable survival outcomes remains uncertain because of their distinct pharmacological mechanisms. A comprehensive synthesis of the available evidence is therefore warranted.

In this study, we performed a systematic review and meta-analysis to evaluate the association between RASi use and survival outcomes in patients with lung cancer. We further examined whether the associations with survival differed between ACEi and ARB, explored potential sources of heterogeneity through predefined subgroup and sensitivity analyses, and assessed the robustness of the pooled evidence.

## Methods

### Protocol and guideline

The protocol for this systematic review and meta-analysis was prospectively registered in the International Prospective Register of Systematic Reviews (PROSPERO; CRD420251053671). The review was conducted and reported in accordance with the Preferred Reporting Items for Systematic Reviews and Meta-Analyses (PRISMA) 2020 statement (Page et al., 2021).

### Data sources and study selection

A comprehensive literature search was conducted in PubMed and Embase to identify studies evaluating the association between RASi use and survival outcomes in patients with lung cancer. The search strategy combined Medical Subject Headings (MeSH) and Emtree terms with relevant free-text keywords related to lung cancer, the renin–angiotensin system, and specific RASi agents. No restrictions on publication year were applied. The final search was completed on 6 July 2026. In addition, the reference lists of eligible articles and relevant reviews were manually screened to identify potentially eligible studies. The complete search strategy is provided in Supplementary Table 1.

Studies were eligible if they (1) evaluated the association between RASi (ACEi and/or ARB) use and survival outcomes in patients with lung cancer; (2) reported hazard ratios (HRs) with corresponding 95% confidence intervals (CIs), or provided sufficient data for their estimation; and (3) included a comparator group of patients not receiving RASi. Eligible study designs included prospective or retrospective cohort studies, case-control studies, and post hoc analyses of clinical trials. Studies were excluded if they were preclinical studies, case reports, reviews, editorials, conference abstracts without sufficient data, studies involving non-lung cancer populations, or studies lacking a non-RASi comparator group.

After duplicate records were removed using EndNote, two reviewers independently screened titles and abstracts for eligibility. Full texts of potentially relevant articles were subsequently assessed independently for inclusion. Disagreements were resolved through discussion with a third reviewer until consensus was reached.

### Data extraction and quality assessment

Data extraction was performed by one reviewer and independently verified by a second reviewer using a standardized data collection form. Discrepancies were resolved by consensus. Extracted data included first author, publication year, country, study design, sample size, RASi exposure (ACEi and/or ARB), proportion of RASi users, lung cancer histology and stage, anticancer treatment modality, and HRs with corresponding 95% CIs for OS and PFS. When both adjusted and unadjusted estimates were reported, the most fully adjusted HR was extracted. If separate HRs were reported for ACEi and ARB, these estimates were extracted individually for the corresponding subgroup analyses.

Methodological quality was assessed independently by two reviewers using the Newcastle–Ottawa Scale (NOS) (Stang, 2010). Studies scoring 7–9 points were considered high quality, 5–6 points moderate quality, and ≤4 points low quality. Disagreements were resolved by consensus. Study quality was evaluated to facilitate interpretation of the findings but was not used as an exclusion criterion.

### Statistical analysis

HRs with corresponding 95% CIs were pooled using random-effects models with the Sidik–Jonkman estimator for between-study variance (τ^2^), given the anticipated clinical and methodological heterogeneity among observational studies. Statistical heterogeneity was assessed using Cochran’s *Q* test and quantified with the *I* ^*2*^ statistic, with *I* ^*2*^ values > 50% considered indicative of substantial heterogeneity.

Prespecified subgroup analyses were conducted according to RASi class (ACEi vs. ARB), anticancer treatment modality (chemotherapy or immunotherapy), and geographic region (Asia, Europe, and North America).

Sensitivity analyses were performed to evaluate the robustness of the findings and included leave-one-out analyses, sequentially excluding each study to assess its influence on the pooled estimate, and large-study restricted analyses including only studies with a standard error less than or equal to the median. When evidence of small-study effects was identified, precision-effect test and precision-effect estimate with standard error (PET-PEESE) regression analyses were performed to obtain bias-adjusted estimates.

Potential small-study effects were assessed by visual inspection of funnel plots together with Egger’s linear regression test when at least 10 effect estimates were available. Statistical significance for Egger’s test was defined as *P* < 0.10. When evidence of small-study effects was identified, PET-PEESE regression was performed as a sensitivity analysis to examine the robustness of the pooled estimate to potential small-study effects.

All statistical analyses were performed using R software (version 4.5.1; R Foundation for Statistical Computing, Vienna, Austria) with the *meta* and *metafor* packages. All statistical tests were two-sided, and *P* < 0.05 was considered statistically significant unless otherwise specified.

## Result

### Study selection

The literature search identified 2,745 records, of which 2,308 remained after duplicate removal. Following title and abstract screening, 31 articles were retrieved for full-text review. After assessment against the predefined eligibility criteria, 26 studies were included in the final meta-analysis (Wilop et al., 2009; Hsieh et al., 2025; Kichenadasse et al., 2021; Holmes et al., 2013; Aydiner et al., 2015; Miao et al., 2016; Menter et al., 2017; Zhu et al., 2017; Cui et al., 2019; Wei et al., 2019; Kocher et al., 2021; Kostine et al., 2021; Miura et al., 2021; Pereira et al., 2021; Tozuka et al., 2021; Chiang et al., 2023; Zhang et al., 2023; Ma et al., 2024; Rousseau et al., 2024; Brinzevich et al., 2025; Choi et al., 2025; Gobbini et al., 2025; Sever et al., 2025; Efil et al., 2026; Ikoma et al., 2026; Mutlu Ozkan et al., 2026). The study selection process is illustrated in Figure 1.

**FIGURE 1:**
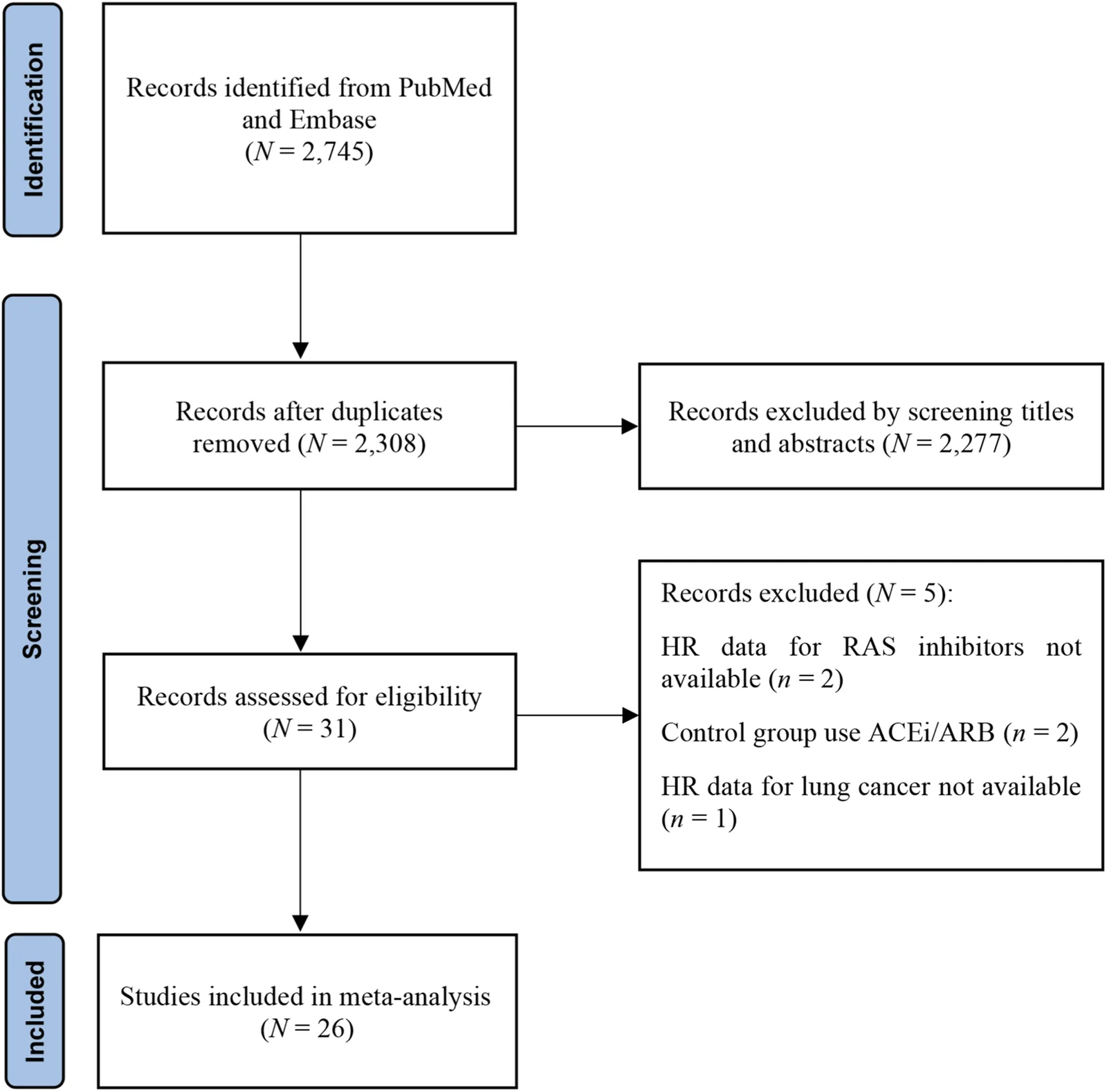
PRISMA 2020 flow diagram of study selection.

### Characteristics of the included studies

The 26 included studies comprised 25 retrospective cohort studies and one post hoc analysis, involving a total of 403,423 patients with lung cancer. Sample sizes ranged from 107 to 347,062 patients. Sixteen studies were conducted in Asia, six in Europe, and three in North America. The proportion of RASi users ranged from 11.7% to 50.0%. Patients received a variety of anticancer therapies, including immune checkpoint inhibitors, platinum-based chemotherapy, and epidermal growth factor receptor tyrosine kinase inhibitors (EGFR-TKIs). The details of included studies are summarized in Table 1. Study quality was generally high, with 25 studies rated as high quality and one as moderate quality according to the NOS. Detailed study characteristics are presented in Supplementary Table 2.

**Table 1:** Characteristics of studies included in meta–analysis.

Study	Design	Country	Users/Sample size (%)	RAS inhibitors used	Histology	Stage	Treatment	Population in control group	HR for OS (95% CI)	HR for PFS (95% CI)
Wilop et al. (2009)	RS	Germany	52/287 (18.1)	ACEi ARB	NSCLC	IIIB/IV	First-line platinum-based chemotherapy	RAS inhibitor non-users	0.56 (0.33–0.95)	NR
Holmes et al. (2013)	RS	Canada	1,256/3,185 (39.4)	ACEi ARB	Mixed	I–IV	NR	Antihypertensive drug non-users	1.11 (1.03–1.21)	NR
Aydiner et al. (2015)	RS	Turkey	37/117 (31.6)	ACEi ARB	NSCLC	Metastatic	Chemotherapy, targeted therapy	Patients free of RAS inhibitors	0.71 (0.53–0.94)	NR
Miao et al. (2016)	RS	China	52/301 (17.3)	ACEi ARB	NSCLC	I–IV	First-line platinum-based chemotherapy	Patients free of RAS inhibitors	0.80 (0.60–1.06)	0.74 (0.61–0.90)
Menter et al. (2017)	RS	United States	273 /1,465 (18.6)	ACEi ARB	NSCLC	IIIB or IV	Carboplatin and paclitaxel	Patients free of RAS inhibitors	0.73 (0.61–0.88)	NR
			78/348 (22.4)				Carboplatin and paclitaxel with bevacizumab		0.79 (0.52–1.21)	NR
Zhu et al. (2017)	RS	China	143/1,220 (11.7)	ACEi ARB	Mixed	I–IIIa	NR	Non-hypertension patients	0.74 (0.53–1.02)	0.71 (0.49–1.02)
Cui et al. (2019)	RS	China	NR/824	ACEi	Mixed	I–IV	Chemotherapy, radiotherapy, or surgery	Antihypertensive drug non-users	0.84 (0.61–1.16)	NR
				ARB					0.74 (0.54–1.03)	NR
Wei et al. (2019)	RS	China	217/678 (32.0)	ACEi ARB	Mixed	NR	Chemotherapy and others	RAS inhibitor non-users	NR	0.63 (0.40–0.98)
Kichenadasse et al. (2021)	*Post hoc* analysis	Global	358/1,547 (23.1)	ACEi ARB	NSCLC	Advanced	First- or later-line immunotherapy (Atezolizumab)	RAS inhibitor non-users	0.97 (0.77–1.23)	1.05 (0.86–1.28)
									0.78 (0.46–1.30)	0.78 (0.51–1.19)
									0.71 (0.42–0.96)	0.88 (0.71–1.08)
									0.77 (0.41–1.45)	1.14 (0.70–1.86)
Kocher et al. (2021)	RS	Austria	100/444 (22.5)	ACEi ARB	NSCLC	NR	First-line platinum-based palliative chemotherapy	RAS inhibitor non-users	0.59 (0.35–1.00)	0.77 (0.59–0.99)
Kostine et al. (2021)	RS	France	NR/150	ACEi ARB	NSCLC	Advanced	Immunotherapy (mainly ≥ second-line)	RAS inhibitor non-users	0.78 (0.51–1.19)	1.04 (0.69–1.57)
Miura et al. (2021)	RS	Japan	40/300 (13.3)	ARB	NSCLC	Advanced	First- or later-line immunotherapy	RAS inhibitor non-users	0.69 (0.31–1.50)	NR
Pereira et al. (2021)	RS	Portugal	20/112 (17.9)	ACEi	NSCLC	III/IV	Immunotherapy	RAS inhibitor non-users	0.75 (0.39–1.32)	0.87 (0.46–1.65)
			15/107 (14.0)	ARBs					0.44 (0.19–1.02)	0.40 (0.17–0.93)
Tozuka et al. (2021)	RS	Japan	37/256 (14.5)	ACEi ARB	NSCLC	Advanced	Second- or later-line immunotherapy	RAS inhibitor non-users	0.71 (0.45–1.11)	0.65 (0.44–0.98)
Chiang et al. (2023)	RS	China	95/337 (28.2)	ACEi ARB	Mixed	Advanced	Immunotherapy	RAS inhibitor non-users	0.36 (0.23–0.57)	0.55 (0.38–0.79)
Zhang et al. (2023)	RS	China	62/208 (29.8)	ACEi	SCLC	Advanced	First- or later-line immunotherapy (± chemotherapy)	RAS inhibitor non-users	0.82 (0.53–1.27)	1.14 (0.82–1.59)
Ma et al. (2024)	RS	China	38/124 (30.6)	ACEi ARB	Mixed	Advanced	First- or later-line immunotherapy (± chemotherapy)	RAS inhibitor non-users	0.39 (0.11–1.40)	0.37 (0.17–0.79)
Rousseau et al. (2024)	RS	France	13,882/38,176 (36.4%)	ACEi ARB	NSCLC	Advanced	First-line immunotherapy (Pembrolizumab)	RAS inhibitor non-users	1.01 (0.98–1.04)	NR
Brinzevich et al. (2025)	RS	United States	1,101/3,739 (29.4%)	ACEi ARB	NSCLC	Stage IV	First- or second-line immunotherapy	RAS inhibitor non-users	0.95 (0.88–1.04)	NR
Choi et al. (2025)	RS	South Korea	173,531/347,062 (50.0%)	ARB	Mixed	NR	NR	Calcium channel blocker users	0.79 (0.75–0.83)	NR
Gobbini et al. (2025)	RS	France	183/753 (24.3%)	ACEi ARB	NSCLC	IIIB/IV/recurrent	Immunotherapy (Nivolumab)	RAS inhibitor non-users	0.78 (0.65–0.93)	0.86 (0.71–1.03)
Hsieh et al. (2025)	RS	China	63/238 (26.5%)	ACEi ARB	NSCLC	IIIA/IIIB/IV	EGFR-TKI (gefitinib/erlotinib)	RAS inhibitor and β-blocker non-users	0.62 (0.44–0.86)	0.79 (0.57–1.08)
Sever et al. (2025)	RS	Turkey	107/300 (35.7)	ACEi ARB	NSCLC	Metastatic	First-line chemotherapy, immunotherapy	RAS inhibitor non-users	0.72 (0.55–0.95)	NR
Efil et al. (2026)	RS	Turkey	94/286 (32.9%)	ACEi ARB	Mixed	IIIB-IIIC/IV	Immunotherapy ± chemotherapy	RAS inhibitor non-users	0.58 (0.43–0.80)	0.76 (0.57–1.00)
Ikoma et al. (2026)	RS	Japan	25/89 (28.1%)	ARB	NSCLC	IIIB/IV/recurrent	First-line immunotherapy	ARB non-users	NR	1.06 (0.64–1.76)
			78/257 (30.4%)				First-line chemo-immunotherapy		NR	0.70 (0.53–0.93)
Mutlu Ozkan et al. (2026)	RS	Turkey	288/614 (46.9%)	ACEi ARB	NSCLC	Metastatic	Second-line immunotherapy (Nivolumab)	RAS inhibitor non-users	0.60 (0.46–0.79)	0.75 (0.59–0.94)

Abbreviation: RAS, renin–angiotensin system; ACEi, angiotensin–converting enzyme inhibitors; ARB, angiotensin receptor blockers; RS, retrospective cohort study; NSCLC, non–small cell lung cancer; SCLC, small cell lung cancer, HR, hazard ratio, OS, overall survival; PFS, progression–free survival; NR, not reported.

### RASi use and survival outcomes

Cui et al. (2019) and Pereira et al. (2021) reported HRs stratified by RASi type, while Menter et al. (2017) and Ikoma et al. (2026) presented HRs stratified by concomitant anticancer therapies, and Kichenadasse et al. (2021) provided HRs according to each trial. Accordingly, these studies were treated as separate datasets in the pooled analysis. Overall, 30 cohorts contributed data for OS and 21 cohorts for PFS.

For OS, 24 studies comprising 30 cohorts (402,399 patients) were included. The pooled analysis demonstrated that RASi use was associated with improved OS (HR = 0.76, 95% CI 0.70–0.83; Figure 2), with substantial between-study heterogeneity (*I* ^*2*^ = 82.9%).

**FIGURE 2:**
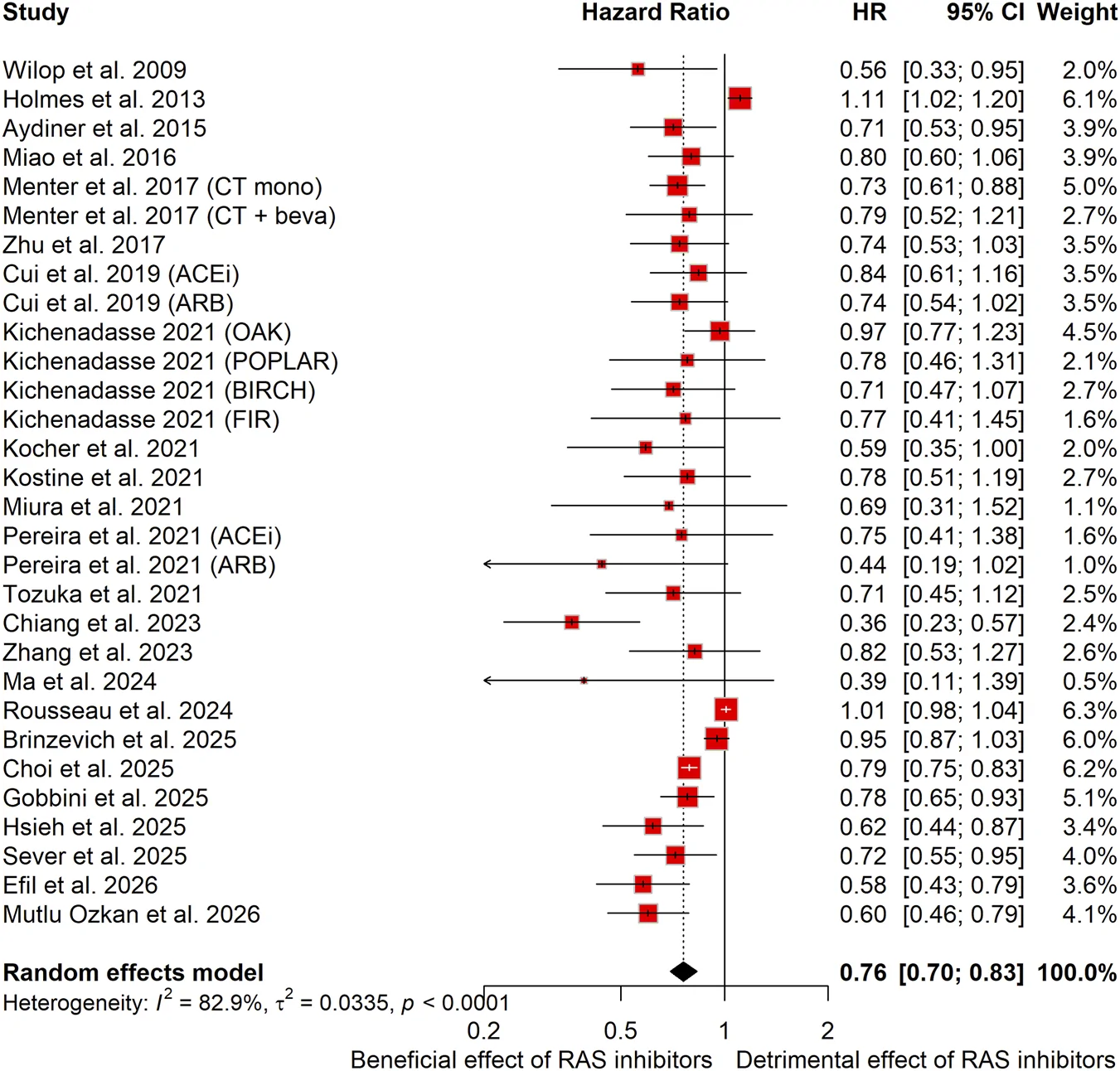
Forest plot of overall survival for RASi users versus non-users.

For PFS, 16 studies comprising 21 cohorts (7,620 patients) were included. RASi use was also associated with improved PFS (HR = 0.79, 95% CI 0.70–0.89; Figure 3), with moderate heterogeneity (*I* ^*2*^ = 40.9%).

**FIGURE 3:**
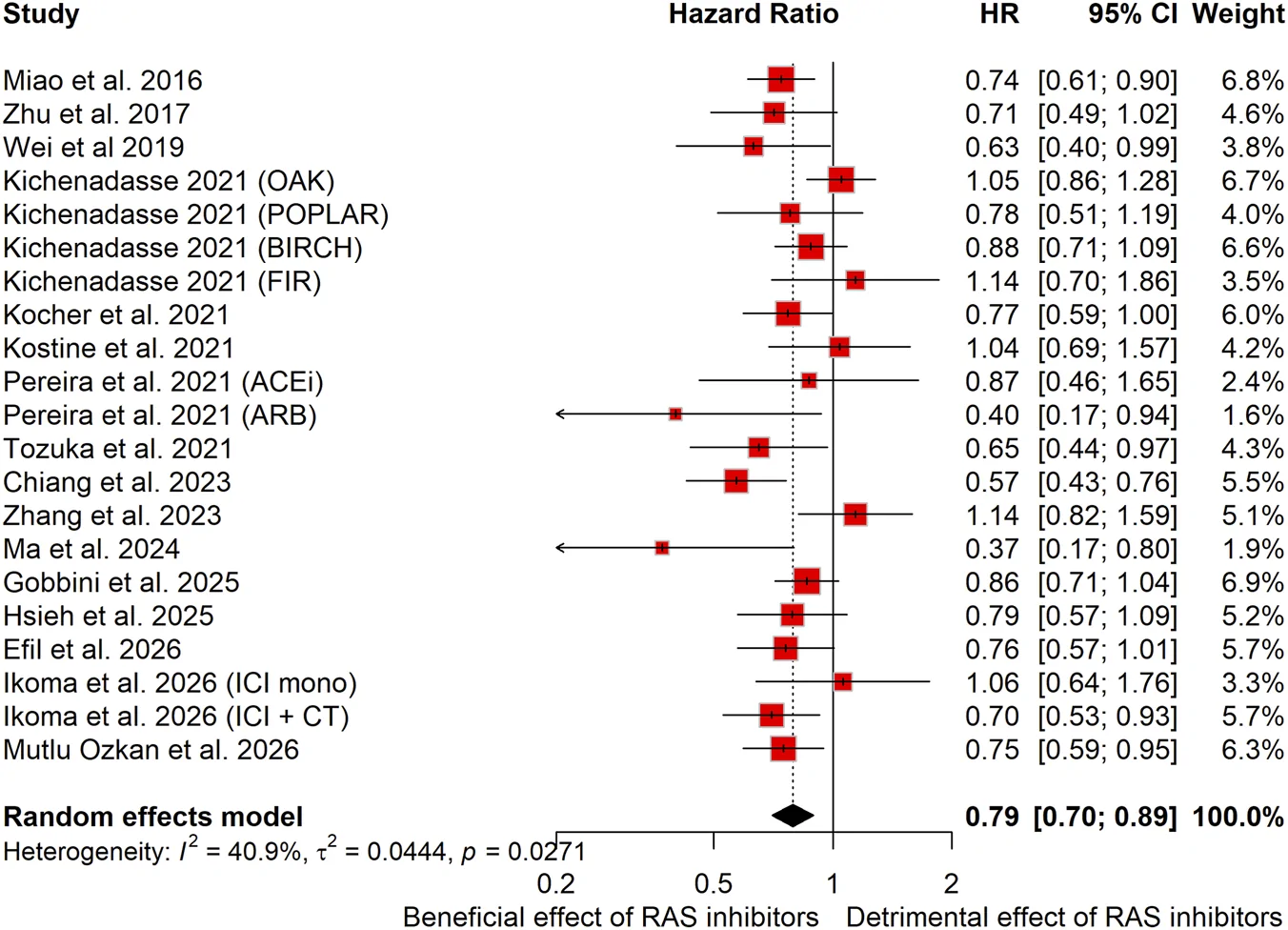
Forest plot of progression-free survival for RASi users versus non-users.

### Subgroup analysis

Subgroup analyses were performed to explore potential sources of heterogeneity and to evaluate the consistency of RASi-associated survival benefits across different clinical and demographic contexts. Results are summarized in Figure 4.

**FIGURE 4:**
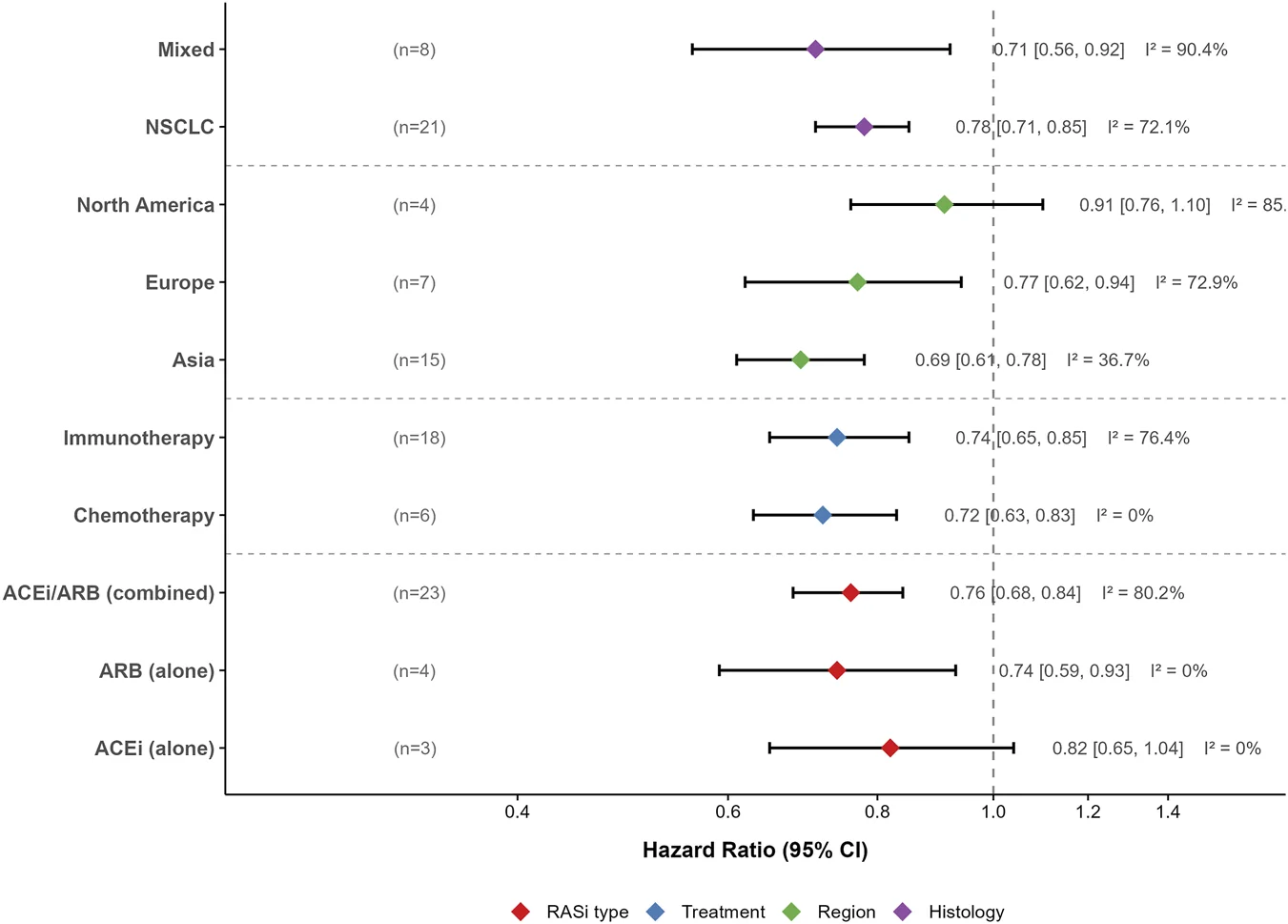
Summary of subgroup analyses for overall survival.

For OS, both ACEi and ARB were associated with numerically lower mortality compared with non-use. The pooled HR was 0.82 (95% CI 0.65–1.04; *I* ^*2*^ = 0%) for ACEi and 0.74 (95% CI 0.59–0.93; *I* ^*2*^ = 0%) for ARB. Studies evaluating combined ACEi/ARB exposure showed a pooled HR of 0.76 (95% CI 0.68–0.84; *I* ^*2*^ = 80.2%) (Supplementary Figure 1). Subgroup analyses for PFS according to RASi class were limited by the small number of available studies (Supplementary Figure 2).

Across treatment modalities, RASi use was associated with improved OS in both chemotherapy-treated patients (HR = 0.72, 95% CI 0.63–0.83; *I* ^*2*^ = 0%) and immunotherapy-treated patients (HR = 0.74, 95% CI 0.65–0.85; *I* ^*2*^ = 76.4%), without evidence of a significant subgroup difference (Supplementary Figure 3). Similar findings were observed for PFS (Supplementary Figure 4).

When stratified by geographic region, the pooled HRs for OS were 0.69 (95% CI 0.61–0.78) in Asia, 0.77 (95% CI 0.62–0.94) in Europe, and 0.91 (95% CI 0.76–1.10) in North America (Supplementary Figure 5). Comparable findings were observed for PFS across geographic subgroups (Supplementary Figure 6).

When stratified by histological subtype, RASi use was associated with improved OS in studies including patients with non-small cell lung cancer (HR = 0.78, 95% CI 0.71–0.85) and in studies enrolling mixed histological populations (HR = 0.71, 95% CI 0.56–0.92), with no significant subgroup difference (Supplementary Figure 7). Similar results were observed for PFS (Supplementary Figure 8).

### Meta-regression analysis

To explore potential sources of the substantial heterogeneity observed for OS (*I* ^*2*^ = 82.9%), we performed univariate meta-regression analyses using five prespecified covariates: treatment modality, geographic region, RASi type, publication year, and log-transformed sample size. Log-transformed sample size was significantly associated with the estimated OS effect (HR = 1.045 per unit increase, 95% CI: 1.004–1.088, *P* = 0.032), explaining approximately 17.7% of the between-study variance (adjusted *R* ^2^ = 17.68%). Geographic region did not reach statistical significance as an overall moderator (*P* = 0.068; adjusted *R* ^2^ = 31.08%), although the Global region showed a significantly weaker association than Asia (HR = 1.32, 95% CI: 1.07–1.63, *P* = 0.012). Treatment modality (*P* = 0.55; adjusted *R* ^2^ = −1.74%), RASi type (*P* = 0.88; adjusted *R* ^2^ = −10.90%), and publication year (*P* = 0.42; adjusted *R* ^2^ = −1.26%) were not significantly associated with between-study heterogeneity. Nevertheless, substantial residual heterogeneity remained across the meta-regression models, indicating that the observed variability in treatment effects was likely multifactorial and may also reflect unmeasured differences in patient characteristics, RASi exposure patterns, treatment settings, or healthcare systems. Detailed results are presented in Supplementary Table 3.

### Sensitivity analysis

Leave-one-out analyses demonstrated that sequential exclusion of individual studies did not materially alter the pooled estimates for either OS (HR range, 0.75–0.79; Supplementary Figure 9) or PFS (HR range, 0.78–0.81; Supplementary Figure 10).

For the OS analysis, the median standard error of the log HR was 0.174. The large-study restricted analysis included 15 studies with a standard error ≤0.174 and yielded a pooled HR of 0.82 (95% CI: 0.74–0.90), which remained consistent with the primary analysis (Supplementary Figure 11).

### Publication bias

For OS, visual inspection of the funnel plot suggested asymmetry, and Egger’s regression test indicated evidence of small-study effects (Figure 5). PET-PEESE adjustment attenuated the pooled estimate to the null (HR = 1.00, 95% CI: 0.95–1.05), indicating that the observed OS association was sensitive to adjustment for small-study effects and that its robustness remains uncertain. For PFS, the funnel plot appeared symmetrical, and Egger’s regression test did not indicate significant publication bias (Supplementary Figure 12).

**FIGURE 5:**
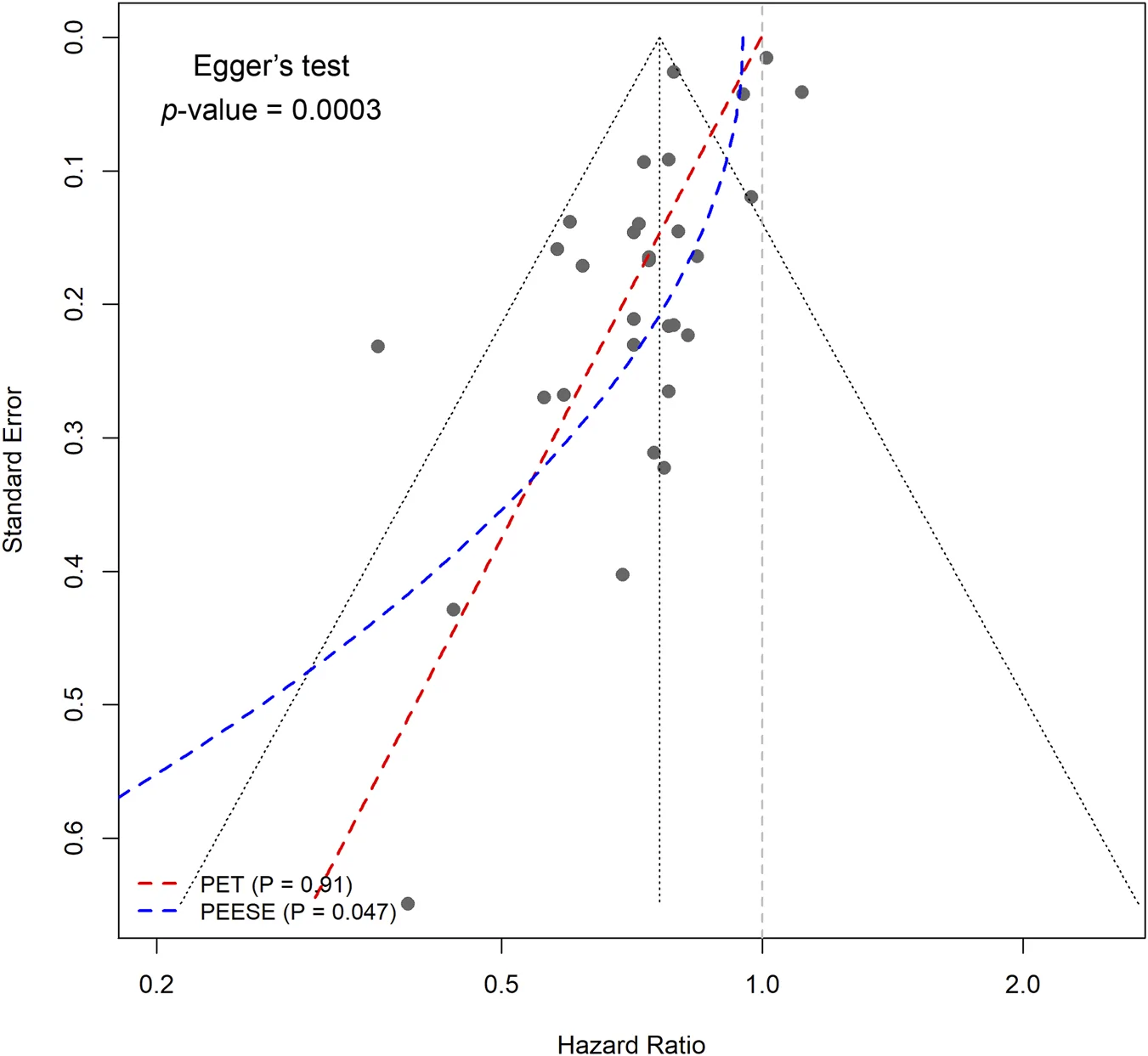
Funnel plot for assessment of publication bias in overall survival.

## Discussion

In this systematic review and meta-analysis of 26 studies involving more than 400,000 patients with lung cancer, RASi use was associated with improved OS and PFS in conventional random-effects analyses. However, the OS association was attenuated to the null after PET-PEESE adjustment for small-study effects, whereas the PFS association was supported by relatively low heterogeneity and no significant evidence of small-study effects. The observed associations were generally directionally consistent across prespecified subgroup analyses; however, substantial heterogeneity and evidence of small-study effects warrant cautious interpretation of the pooled estimates.

Our findings are broadly consistent with previous meta-analyses reporting favorable survival outcomes associated with RASi use in several solid malignancies, including pancreatic, ovarian, and renal cancers (Lin et al., 2025; Wang et al., 2025; Xi et al., 2025). Compared with the existing literature in lung cancer, the present study incorporates substantially more patients and includes studies evaluating contemporary systemic therapies, particularly immune checkpoint inhibitors. Although individual studies have reported inconsistent findings, likely reflecting differences in patient populations, treatment settings, exposure definitions, and analytical methods, the conventional pooled analyses suggest an association between RASi exposure and favorable survival outcomes across diverse clinical settings. However, the attenuation of the OS estimate after PET-PEESE adjustment indicates that the strength and robustness of this association, particularly for OS, remain uncertain.

Several biological mechanisms may explain these observations. Beyond its established role in cardiovascular homeostasis, the RAS has emerged as an important regulator of the tumor microenvironment (Pinter and Jain, 2017). Ang II signaling through AT_1_R promotes angiogenesis, inflammation, extracellular matrix remodeling, and fibrosis, all of which contribute to tumor progression (George et al., 2010; Butcher et al., 2026). Experimental studies have shown that RAS inhibition may normalize tumor vasculature, reduce stromal fibrosis, suppress immunosuppressive myeloid cell populations, and enhance intratumoral CD8^+^ T-cell infiltration, thereby improving drug delivery and antitumor immune responses (Cortez-Retamozo et al., 2013; Gu et al., 2023; Chauhan et al., 2013). These mechanisms provide biological plausibility for the survival advantages observed in patients receiving chemotherapy and immunotherapy.

An important consideration is the apparent discrepancy between the potential increase in lung cancer incidence associated with ACEi exposure and the favorable survival association observed among patients with established lung cancer. These findings address different clinical stages and should therefore not be interpreted as contradictory. The epidemiological and genetic evidence linking ACEi exposure to lung cancer risk primarily concerns cancer development, whereas the present analysis evaluates prognosis after cancer diagnosis. Several mechanisms may contribute to this apparent divergence. ACE inhibition increases bradykinin and substance P levels, which have been proposed to influence processes involved in carcinogenesis (Wu et al., 2023), whereas blockade of Ang II–AT_1_R signaling may exert effects on tumor angiogenesis, stromal remodeling, inflammation, and immune regulation after tumor establishment (Perini et al., 2020). In addition, differences in smoking exposure, treatment duration, indication for RASi use, and baseline comorbidity may contribute to the observed discrepancy. Nevertheless, these mechanistic explanations remain speculative, and the present observational evidence cannot establish that RASi have opposing effects on cancer incidence and prognosis.

Subgroup analyses by RASi type provided some evidence of differential patterns between ACEi and ARB. ARB use was associated with a statistically significant OS benefit (HR = 0.74, 95% CI: 0.59–0.93; *I* ^*2*^ = 0%), whereas ACEi use showed a non-significant trend toward benefit (HR = 0.82, 95% CI: 0.65–1.04; *I* ^*2*^ = 0%). However, the test for subgroup differences was not statistically significant (*P* = 0.800), and the confidence intervals overlapped, precluding a conclusion that ARB is superior to ACEi. The numerically stronger association observed with ARB may nevertheless warrant further investigation given their distinct pharmacological mechanisms. ACEi inhibits Ang II formation while increasing bradykinin and substance P levels, whereas ARB selectively blocks AT_1_R signaling without directly affecting bradykinin metabolism. These mechanistic differences may theoretically result in distinct effects on tumor biology; however, the limited number of studies providing RASi class-specific estimates, particularly for ACEi, and the observational nature of the available evidence preclude definitive conclusions. Thus, the apparent difference between ACEi and ARB should be considered hypothesis-generating rather than evidence of differential clinical efficacy.

The substantial heterogeneity observed in the immunotherapy subgroup (*I* ^*2*^ = 76.4%) warrants particular consideration. Several factors may contribute to this variability. RAS inhibition may influence the tumor microenvironment through multiple mechanisms, including modulation of tumor angiogenesis, vascular function, extracellular matrix remodeling, fibrosis, and immunosuppressive myeloid-cell populations, potentially affecting the activity of immune checkpoint blockade (Shen et al., 2023; Pinter and Jain, 2017). However, the magnitude of these effects may depend on tumor histology, baseline immune and stromal characteristics, and the degree of RAS activation. In addition, the included immunotherapy cohorts differed substantially in treatment setting and regimen, including the type of immune checkpoint inhibitor, treatment line, and use of concomitant chemotherapy. Such clinical and pharmacological differences may modify the interaction between RAS inhibition and antitumor immunity and contribute to the observed heterogeneity. Although pharmacological interactions between RASi and immune checkpoint inhibitors are biologically plausible at the tumor-microenvironment level, the available clinical evidence is insufficient to establish a specific pharmacokinetic or pharmacodynamic drug–drug interaction. Therefore, the high heterogeneity observed in this subgroup should be interpreted primarily in the context of differences in tumor biology, treatment regimens, patient characteristics, and concomitant therapies rather than as evidence of a specific drug–drug interaction.

Meta-regression analyses further explored potential sources of heterogeneity in OS. Log-transformed sample size was significantly associated with the estimated OS effect and explained approximately 17.7% of the between-study variance, suggesting that study size contributed to variability in the reported treatment effects and was consistent with the small-study effects identified in the publication-bias analyses. Geographic region showed a trend toward an overall moderating effect (*P* = 0.068; adjusted *R* ^2^ = 31.08%), although the overall association did not reach conventional statistical significance. Notably, the Global population showed a significantly weaker association than the Asian population (HR = 1.32, 95% CI: 1.07–1.63; *P* = 0.012). In contrast, treatment modality, RASi type, and publication year were not significantly associated with between-study heterogeneity. Importantly, substantial residual heterogeneity remained across models, indicating that the observed variability was likely multifactorial. Differences in baseline patient characteristics, tumor stage, treatment line, RASi exposure patterns, duration of therapy, concomitant medications, and healthcare settings may have contributed to the remaining heterogeneity. Therefore, no single clinical or methodological factor adequately explains the heterogeneity observed in the pooled OS analysis, and the pooled estimate should be interpreted with appropriate caution.

An additional consideration is the substantial contribution of the large population-based study by Choi et al. (2025), which accounted for approximately 86% of the total pooled population. Although its large sample size gives this study substantial statistical weight, the influence of a single large cohort should be interpreted in conjunction with differences in comparator selection across studies. Choi et al. used calcium channel blocker users as an active comparator, whereas most other studies used RASi non-users as the reference group. These comparator strategies address different sources of confounding and yield effect estimates with different clinical interpretations. Comparisons with RASi non-users may be influenced by differences in hypertension status and comorbidity burden, whereas an active-comparator design may improve comparability among patients receiving antihypertensive treatment but introduces the potential influence of the comparator drug itself. Therefore, heterogeneity in comparator selection represents an additional limitation when synthesizing the available observational evidence and may contribute to residual between-study heterogeneity.

The potential clinical implications of these findings deserve careful consideration. RASi are inexpensive, widely available, and have well-established cardiovascular indications. For patients with lung cancer who have an established indication for ACEi or ARB therapy, our findings provide no evidence that RASi use is associated with adverse oncologic outcomes and suggest a potentially favorable association with survival in conventional analyses. However, the substantial heterogeneity across studies, the observational nature of the available evidence, and the attenuation of the OS association to the null after PET-PEESE adjustment substantially limit the strength of this inference. In particular, the PET-PEESE estimate (HR ≈ 1.00) indicates that the apparent OS benefit may be sensitive to small-study effects. Therefore, the present findings do not support initiating RASi solely for anticancer purposes outside established cardiovascular indications. Rather, RASi should be regarded as a hypothesis-generating repurposing strategy that warrants prospective evaluation, particularly in well-defined patient populations receiving contemporary lung cancer therapies.

Several limitations should also be acknowledged. First, almost all included studies were retrospective observational studies, precluding causal inference and leaving the possibility of residual confounding despite multivariable adjustment. Important sources of bias, including confounding by indication, immortal time bias, and differences in baseline comorbidity, cannot be completely excluded. Notably, none of the included studies explicitly reported using a landmark analysis to address immortal time bias, and no study used time-dependent Cox regression as its primary analytical approach to account for time-varying RASi exposure. Although one study considered but did not apply a time-dependent model because RASi exposure was considered stable during follow-up, the absence of consistent approaches to account for time-dependent exposure limits our ability to exclude immortal time bias. This issue may have contributed to an overestimation of the apparent benefit of RASi in studies in which exposure classification involved an immortal period.

Second, substantial heterogeneity was observed for several analyses, particularly OS. Although meta-regression identified study size as a significant contributor and suggested a potential role for geographic differences, substantial residual heterogeneity remained, indicating that important sources of variability could not be fully captured by the available study-level data. Differences in patient populations, tumor stage, histology, systemic therapies, RASi exposure patterns, and healthcare settings may have contributed to the remaining heterogeneity.

Third, we were unable to perform a meaningful subgroup analysis according to tumor stage. The included studies encompassed heterogeneous patient populations, ranging from early-stage disease to advanced or metastatic disease. Moreover, stage reporting was inconsistent across studies, with some studies enrolling patients across broad stage ranges (e.g., I–IV or IIIA–IV), others focusing predominantly on advanced disease, and several studies providing insufficient stage information. These differences precluded a reliable and clinically meaningful classification into early-versus advanced-stage populations using the available aggregate data. Given the potential influence of tumor burden, treatment setting, and disease biology on survival outcomes, differences in stage distribution may have contributed to the observed heterogeneity. Future studies should evaluate the prognostic association of RASi according to clearly defined and consistently reported disease stages.

Fourth, information regarding treatment duration, cumulative dose, medication adherence, and molecular characteristics, including EGFR and ALK alterations, was inconsistently reported, limiting more detailed subgroup analyses. Additionally, evidence of funnel plot asymmetry and small-study effects was identified for OS. Although PET-PEESE adjustment attenuated the pooled estimate to the null, the association remained statistically significant in the large-study restricted analysis. This discrepancy indicates that the magnitude and robustness of the observed OS association are uncertain and may have been influenced by small-study effects. Importantly, PET-PEESE adjustment cannot establish that small-study effects are attributable specifically to publication bias, and the possibility of a true association cannot be excluded.

Finally, although PubMed and Embase provide broad coverage of biomedical and pharmacological literature, the search was not extended to other databases such as Web of Science or the Cochrane Library, and the possibility that additional eligible studies were missed cannot be completely excluded.

Future research should focus on determining whether the observed associations are causal rather than confounded. Well-designed prospective studies and randomized clinical trials are needed to evaluate RAS inhibition as an adjunct to modern systemic therapies, particularly immune checkpoint inhibitors. Future observational studies should also consider active-comparator designs, clearly defined exposure windows, and time-varying exposure analyses where appropriate to minimize confounding by indication and immortal time bias. In parallel, translational studies should investigate biomarkers capable of identifying patients most likely to benefit from RAS blockade and further elucidate the mechanisms through which modulation of the RAS influences the tumor microenvironment and therapeutic response.

## Conclusion

In conventional random-effects analyses, RASi use was associated with improved OS and PFS in patients with lung cancer across a broad range of clinical settings. However, the OS association was attenuated to the null after PET-PEESE adjustment for small-study effects, whereas the PFS association was supported by lower between-study heterogeneity and no significant evidence of small-study effects. Given the predominantly observational nature of the evidence and the potential for residual confounding, immortal time bias, and small-study effects, these findings should be considered hypothesis-generating rather than practice-changing. Prospective studies and randomized clinical trials are required to determine whether RAS inhibition confers causal survival benefits and to define its potential role as an adjunctive strategy in the treatment of lung cancer.

## Data Availability

The original contributions presented in the study are included in the article/Supplementary Material, further inquiries can be directed to the corresponding author.
