# Supplementary material for: Renin–angiotensin system inhibitor use and survival outcomes in lung cancer: a systematic review and meta-analysis
Source: Front Pharmacol. 2026 Sep 21;17:1953204. doi: 10.3389/fphar.2026.1953204 (PMC13635204; doi:10.3389/fphar.2026.1953204)
Supplement: Supplementary file 1 [file Table1.docx]

**Supplementary Material**

**Renin–angiotensin system inhibitor use and survival in lung cancer: a systematic review and meta-analysis**

**Supplementary Table 1.** Details of search strategy.

**Supplementary Table 2.** Quality assessment of included studies through the modified Newcastle-Ottawa Scale.

**Supplementary Table 3.** Meta-regression analysis of factors contributing to the heterogeneity among the included studies.

**Supplementary Figure 1.** Forest plot of overall survival by RASi type.

**Supplementary Figure 2.** Forest plot of progression-free survival by RASi type.

**Supplementary Figure 3.** Forest plot of overall survival by treatment modality.

**Supplementary Figure 4.** Forest plot of progression-free survival by treatment modality.

**Supplementary Figure 5.** Forest plot of overall survival by geographic region.

**Supplementary Figure 6.** Forest plot of progression-free survival by geographic region.

**Supplementary Figure 7.** Forest plot of overall survival by histology.

**Supplementary Figure 8.** Forest plot of progression-free survival by histology.

**Supplementary Figure 9.** Leave-one-out sensitivity analysis for overall survival.

**Supplementary Figure 10.** Leave-one-out sensitivity analysis for progression-free survival.

**Supplementary Figure 11.** Forest plot of large-study restricted analysis for overall survival.

**Supplementary Figure 12.** Funnel plot for assessment of publication bias in progression-free survival.

**Table S1.** Details of searching strategy.

| **Database** | **Search strategy** |
| --- | --- |
| ***PubMed*** | (((((“Angiotensin-Converting Enzyme Inhibitors”[Mesh] OR “Angiotensin-Converting Enzyme Antagonist*” OR “ACE Inhibitor*” OR “Angiotensin I-Converting Enzyme Inhibitor*” OR “Angiotensin-Converting Enzyme Inhibitor*”)) OR ((“Receptors, Angiotensin”[Mesh] OR “Angiotensin Receptor Blocker*” OR “Angiotensin Receptor Antagonist*” OR “Angiotensin II Receptor Antagonist*” OR “Angiotensin II Receptor Blocker*”))) OR ((“Renin-Angiotensin System”[Mesh] OR “Renin-Angiotensin-Aldosterone System”)))) AND ((((“Lung Neoplasms”[Mesh]) OR (“Lung Cancer”)) OR ((“Carcinoma, Non-Small-Cell Lung”[Mesh]) OR (“Non-Small Cell Lung Cancer”))) OR ((“Small Cell Lung Carcinoma”[Mesh]) OR (“Small Cell Lung Cancer”))) |
| ***Embase*** | ('renin-angiotensin system'/exp OR 'angiotensin-converting enzyme inhibitors' OR 'angiotensin-converting enzyme antagonist*' OR 'ace inhibitor*' OR 'angiotensin i-converting enzyme inhibitor*' OR 'angiotensin-converting enzyme inhibitor*' OR captopril OR enalapril OR benazepril OR fosinopril OR perindopril OR ramipril OR 'angiotensin receptor blocker*' OR 'angiotensin receptor antagonist*' OR 'angiotensin ii receptor antagonist*' OR 'angiotensin ii receptor blocker*' OR losartan OR irbesartan OR valsartan OR telmisartan OR olmesartan OR candesartan) AND ('lung cancer'/exp OR 'non-small cell lung cancer' OR 'small cell lung cancer') |

**Supplementary Table 2** Quality assessment of included studies through the modified Newcastle-Ottawa Quality Assessment Scale (NOS).

| **Study** | **Selection (0-4)** | **Comparability (0-2)** | **Outcome (0-3)** | **Total score (0-9)** | **Quality** |
| --- | --- | --- | --- | --- | --- |
| Wilop 2009 | 4 | 2 | 3 | 9 | High |
| Holmes 2013 | 4 | 1 | 3 | 8 | High |
| Aydiner 2015 | 3 | 1 | 3 | 7 | High |
| Miao 2016 | 3 | 2 | 3 | 8 | High |
| Menter 2017 | 4 | 2 | 3 | 9 | High |
| Zhu 2017 | 4 | 2 | 3 | 9 | High |
| Cui 2019 | 4 | 2 | 3 | 9 | High |
| Wei 2019 | 3 | 1 | 3 | 7 | High |
| Kichenadasse 2021 | 4 | 2 | 3 | 9 | High |
| Kocher 2021 | 4 | 2 | 3 | 9 | High |
| Kostine 2021 | 4 | 2 | 3 | 9 | High |
| Miura 2021 | 3 | 2 | 3 | 8 | High |
| Pereira 2021 | 2 | 1 | 2 | 5 | Moderate |
| Tozuka 2021 | 4 | 2 | 3 | 9 | High |
| Chiang 2023 | 4 | 2 | 3 | 9 | High |
| Zhang 2023 | 3 | 2 | 3 | 8 | High |
| Ma 2024 | 3 | 2 | 2 | 7 | High |
| Rousseau 2024 | 4 | 2 | 3 | 9 | High |
| Brinzevich 2025 | 4 | 2 | 3 | 9 | High |
| Choi 2025 | 4 | 1 | 3 | 8 | High |
| Gobbini 2025 | 4 | 2 | 3 | 9 | High |
| Hsieh 2025 | 3 | 2 | 3 | 8 | High |
| Sever 2025 | 4 | 2 | 3 | 9 | High |
| Efil 2026 | 4 | 2 | 3 | 9 | High |
| Ikoma 2026 | 3 | 2 | 3 | 8 | High |
| Mutlu Ozkan 2026 | 4 | 2 | 3 | 9 | High |

**Supplementary Table 3.** Meta-regression analysis of factors contributing to the heterogeneity among the included studies.

| **Variable** | **HR** | **95% CI** | ***P*** |
| --- | --- | --- | --- |
| **Treatment (*I^2^* = 79.93%, adjusted *R^2^* = -1.74%)** | | | |
| Chemotherapy ± targeted therapy (reference) | 0.712 | 0.578–0.878 | 0.003 |
| treat_2（Immunotherapy ± chemotherapy) | 1.059 | 0.830–1.352 | 0.632 |
| treat_3（Mixed) | 1.107 | 0.737–1.663 | 0.611 |
| treat_4（Not reported) | 1.248 | 0.914–1.703 | 0.155 |
| treat_5（Targeted therapy) | 0.871 | 0.505–1.501 | 0.605 |
| **Region (*I^2^* = 61.01%, adjusted *R^2^* = 31.08%)** | | | |
| Asia (reference) | 0.695 | 0.617–0.782 | <0.001 |
| region_2（Europe) | 1.210 | 0.910–1.609 | 0.181 |
| region_3（Global) | 1.321 | 1.070–1.633 | 0.012 |
| region_4（North America) | 1.146 | 0.924–1.420 | 0.204 |
| **RASi type (*I^2^* = 76.17%, adjusted *R^2^* = -10.90%)** | | | |
| ACEi (reference) | 0.814 | 0.579–1.143 | 0.224 |
| type_2 (ACEi or ARB) | 0.935 | 0.655–1.335 | 0.703 |
| type_3 (ARB) | 0.896 | 0.579–1.388 | 0.611 |
| **Year (*I^2^* = 82.82%, adjusted *R^2^* = -1.26%)** | 0.991 | 0.970–1.013 | 0.418 |
| **logSample (*I^2^* = 83.41%, adjusted *R^2^* = 17.68%)** | 1.045 | 1.004–1.088 | 0.032 |

**Supplementary Figure 1.** Forest plot of overall survival by RASi type.

**Supplementary Figure 2.** Forest plot of progression-free survival by RASi type.

**Supplementary Figure 3.** Forest plot of overall survival by treatment modality.

**Supplementary Figure 4.** Forest plot of progression-free survival by treatment modality.

**Supplementary Figure 5.** Forest plot of overall survival by geographic region.

**Supplementary Figure 6.** Forest plot of progression-free survival by geographic region.

**Supplementary Figure 7.** Forest plot of overall survival by histology.

**Supplementary Figure 8.** Forest plot of progression-free survival by histology.

**Supplementary Figure 9.** Leave-one-out sensitivity analysis for overall survival.

**Supplementary Figure 10.** Leave-one-out sensitivity analysis for progression-free survival.

**Supplementary Figure 11.** Forest plot of large-study restricted analysis for overall survival.

**Supplementary Figure 12.** Funnel plot for assessment of publication bias in progression-free survival.
